# Effect of SILPs on the Vulcanization and Properties of Ethylene–Propylene–Diene Elastomer

**DOI:** 10.3390/polym12061220

**Published:** 2020-05-27

**Authors:** Anna Sowińska, Magdalena Maciejewska, Laina Guo, Etienne Delebecq

**Affiliations:** 1 Institute of Polymer and Dye Technology, Lodz University of Technology, Stefanowskiego Street 12/16, 90-924 Lodz, Poland; magdalena.maciejewska@p.lodz.pl; 2 Hutchinson S.A-Research & Innovation Center, Rue Gustave Nourry BP31, 45120 Châlette sur Loing, France; laina.guo@hutchinson.com (L.G.); etienne.delebecq@hutchinson.com (E.D.)

**Keywords:** ionic liquids, solid supports, SILPs, ethylene–propylene–diene rubber, vulcanization

## Abstract

Ionic liquids (ILs) are increasingly used in elastomer technology due to unique physico-chemical properties, which are stable at the temperature of preparation and during processing of rubber compounds. The latest IL application concept is supported ionic liquid-phase (SILP) materials, where an IL film is immobilized on the solid phase. The main aim of this work was studying the influence of IL immobilized on the surface of solid supports, such as silica and carbon black, on the vulcanization process, mechanical properties, and thermal behavior of ethylene–propylene–diene (EPDM) elastomer. Application of the SILP materials enabled the control of EPDM vulcanization without deterioration of the crosslink density, damping properties, thermal stability, and resistance of the vulcanizates to thermo-oxidative aging. Slight improvements in the tensile strength and hardness of the vulcanizates were observed.

## 1. Introduction

ILs, more specifically room temperature ionic liquids (RTILs), are an innovative generation of organic solvents entirely composed of ions, which have attracted significant interest over the last few years [1,2,3,4]. ILs are organic salts, which are liquid at room temperature or have melting points lower than 100 °C. Since the anionic and the cationic parts of ILs can easily be varied, the properties of these compounds can be tuned for specific purposes. The most important features that determine the use of ILs in elastomer technology are their non-flammability, non-volatility and thermal stability at the temperatures of elastomer processing and vulcanization. ILs are being increasingly used in elastomer composites, as vulcanization activators and accelerators, curing agents, conductive additives, and dispersants of fillers [5,6]. Imidazolium ILs are some of the most popular and have been widely used in various types of polymers, including elastomers [7,8]. The imidazolium cation of the ionic liquid plays an important role in improving an ionic conductivity, the mechanical performance and thermal stability of elastomer composites, as well as the dispersion degree of their ingredients, such as curatives and filler. The dispersion of curatives affects the efficiency of crosslinking and distribution of crosslinks in the elastomer network, whereas uniform distribution of the filler’s particles is responsible for the reinforcing effect. Alkylimidazolium ILs have been widely used as dispersants of carbon fillers, such as carbon black, multi-walled carbon nanotubes, or graphene [9,10,11]. Lei et al. proved that using imidazolium ionic liquid improved the dispersion of silica particles in the styrene–butadiene rubber (SBR) composites and increased the interfacial interactions between SBR and the filler, having an important effect on the performance of vulcanizates [12]. Hydrophobic ILs with bis(trifluoromethanesulfonyl)imide anion were applied to improve the dispersion degree of layered fillers, primarily montmorillonite (MMT) and hydrotalcite [13,14]. A homogeneous dispersion accompanied by exfoliation of the filler layers in the polymer matrix was achieved, which is essential to improve the physicochemical properties of the composites. The positive effect of ILs on the curing characteristics, including the optimal vulcanization time of the rubber compounds, as well as the crosslink density, ability to dampen vibrations, and thermal stability of the vulcanizates has been reported [15,16,17]. 

Maciejewska and Zaborski [15] used alkylimidazolium bis(trifluoromethylsulfonyl)imides with different length alkyl chains to improve the degree of dispersion of the curative particles in the SBR matrix. ILs considerably reduced the time of vulcanization and increased the crosslink density of the vulcanizates, which confirms that ILs can catalyze interface crosslinking reactions. It was postulated that ILs can enhance the solubility of the active sulfurating agent, which is formed during vulcanization, increasing the rate and degree of crosslinking [16]. ILs can adsorb on the filler surface [12] and enhance the interphase interactions between the filler and elastomer. During vulcanization, accelerators can be adsorbed on the surface of fillers. Partial adsorption of the accelerator on the fillers surface reduces the effectiveness of its use, and thus can lead to a reduction in the efficiency of crosslinking [18]. Adsorption of ILs on the filler’s surface can reduce the ability of the filler to adsorb water and curatives, which is crucial for effective and fast vulcanization [16].

The disadvantage resulting from the high activity of ILs in the vulcanization is shortening of the scorch time, which affects the safety of rubber compounds processing [14,15,16,17,19]. Therefore, to overcome this drawback, the activity of ILs should be controlled at each stage of vulcanization. The best solution seems to be blocking the activity of the ionic liquid at the initial stage of vulcanization to ensure safe processing, and then unblocking it to enable rapid vulcanization. This possibility can be provided by supported ionic liquid-phase materials (SILPs).

SILP materials are a new concept where an IL film is immobilized on a solid phase [20]. These materials can be prepared using solid supports with different porosities and chemical structures. In SILP systems, a thin IL film is confined on the surface of a highly porous solid using various methods such as tethering, physisorption, or covalent anchoring of ionic liquid fragments. ILs can be immobilized using many different techniques, e.g., simple impregnation, polymerization, grafting, encapsulation, sol-gel, or pore trapping [20,21]. The preparation of SILP materials using several polymeric and inorganic supports has been the subject of several studies. Various materials, such as carbon materials, alumina, different types of silica, hydrotalcites, as well as metal oxides, have been used as support in the preparation of SILPs, which have mainly been applied in catalysis and separation processes so far [22,23,24,25,26,27]. Considering elastomer composites, the literature shows that ILs are, in most cases, added directly to the rubber during the preparation of the rubber compounds. Only a few works focused on the use of ILs immobilized on the surface of fillers [28,29,30,31]. However, surface modification of fillers with ILs is then used to improve the dispersion of filler particles in the elastomer matrix or to study the effect of ILs on elastomer–filler interactions. Promising results were reported by Fleck et al. for carbon black (CB) modified with 1-allyl-3-methylimidazolium chloride (AMICl) [27]. The effect of AMICl-modified CB on the rheological, mechanical and dielectric properties of SBR was investigated. A strong adsorption of AMICl was indicated, resulting in the improvement of the tensile strength and ionic conductivity of the vulcanizates. Strong interactions between AMICl and CB, resulting in improved dispersion of the filler in the elastomer matrix, were confirmed by Kreyenschulte et al. [29]. AMICl appeared to react with both curatives and double bonds of the rubber, which decreased the crosslink density and thus affected the mechanical properties of the vulcanizates, especially elongation at break. Krainoi et al. applied carbon nanotubes (CNTs) modified by an ionic liquid 1-butyl-3-methylimidazolium bis (trifluoromethylsulphonyl)imide (BMI) in natural rubber (NR) matrix. The NR compounds were prepared by the latex mixing method [28]. Modification of CNTs particles with BMI improved their dispersion in the elastomer matrix. BMI significantly affected the vulcanization kinetics of NR compounds acting as a cure retardant, and consequently, increasing the scorch time of rubber compounds. Additionally, the plasticizing effect of BMI was observed. NR composites containing CNTs modified by BMI showed improved storage modulus and stress at relative elongation of 100% and 300%, as compared with those of the unmodified NR vulcanizate. Incorporation of BMI-modified CNTs enhanced the electrical conductivity of the NR composites by formation of three-dimensional CNT networks in the elastomer matrix. The same ionic liquid was studied by Subramaniam et al. in polychloroprene rubber (CR) composites filled with multi-walled carbon nanotubes (MWCNTs). Addition of BMI-modified MWCNTs increased the tensile modulus and hardness of the CR composites, due to more homogeneous dispersion of the nanotubes in the elastomeric matrix. The use of ionic liquid reduced the torque increment during vulcanization, which was attributed to the plasticizing effect of BMI. A positive influence of the CNTs’ modification with ionic liquid, such as 1-decyl-3-methylimidazolium chloride (DMICl), on their dispersion in the elastomer matrix and electrical conductivity was also confirmed for SBR composites [31]. Owing to its ionic conductivity, DMICl was postulated to act as a bridge for electron transfer between the CNTs improving the conductivity of the elastomer composites. Addition of DMICl-modified CNTs increased the stiffness of the vulcanizates, leading to higher tensile modulus and strength at the similar elongation. The positive effect of modified CNTs on the tensile properties of the vulcanizates was attributed to the heterogenous distribution of crosslinks in the elastomer network. The chemical and physical modification of commonly used fillers is still being examined and this is a new aspect of scientific research. However, to the best of our knowledge, SILP materials have not yet been applied to control the vulcanization process. 

In our previous work, ionic liquid 1-decyl-3-methylimidazolium bromide (DmiBr) was immobilized on the surface of silica, calcium oxide, and carbon black (CB) [32]. Thermal analysis and SEM microscopy were employed to characterize the obtained SILPs and determine the efficiency of the filler immobilization with DmiBr dissolved in acetone. Silica and carbon black exhibited high reactivity towards DmiBr. The efficiency of DmiBr immobilization was higher than 80% for both fillers. In the present work, the influence of SILPs with silica and carbon black on the vulcanization process, mechanical properties, and thermal behavior of EPDM elastomer is studied and discussed. The main aim of this study was using SILPs to ensure the safe processing of rubber compounds at 100 °C and their fast vulcanization at 150 °C. As solid supports for SILPs preparation, common reinforcing fillers were applied, such as CB (also used as the primary filler for tested rubber compounds) and Ultrasil VN3 silica. NanoSiO_2_ silica was used to check whether the particle size and specific surface area of filler would affect the properties of SILPs and their effect on the vulcanization process. 

## 2. Materials and Methods

### 2.1. Materials

EPDM rubber with 8.9% ethylidenenorbornene (ENB) and 58% ethylene was obtained from Exxon Mobil (Vistalon 8600, Irving, TX, USA). Its Mooney viscosity was ML1+4 (125 °C): 81. Carbon black (CB, Spheron S0A; Cabot Corporation, Boston, MA, USA) and calcium carbonate (chalk, Omya BSH, Oftringen, Switzerland) were used as fillers. Microsized zinc oxide (ZnO, Huta Bedzin, Bedzin, Poland) was used to activate the vulcanization process. Mineral oil (Torillis 7200, Total Lubricants, CEDEX, France) was used as the processing oil and plasticizer. Calcium oxide granule (CaO, Kezadol GR, Kettlitz-Chemie, Rennertshofen, Germany) was applied as the desiccant. Stearic acid manufactured by Akzo Nobel (Amsterdam, The Netherlands) was used as the softener and filler-dispersing agent. Rubber compounds were cured using sulfur (Torimex-Chemicals, Lodz, Poland) as a crosslinking agent and a system of accelerators, such as benzothiazole disulfide (MBTS), *N*-cyclohexyl-2-benzothiazolesulfenamide (CBS), zinc bis(dibutyl dithiocarbamate) (ZDBC), and 1,3-diphenylguanidine (DPG), which were provided by Brenntag Polska (Kedzierzyn-Kozle, Poland). *N*-phenyl-*N*-(trichloromethylsulphenyl)-benzene sulphonamide (Vulkalent EC 80, Rhein Chemie, Mannheim, Germany) was applied as a retarder and a replasticizing agent. DmiBr (purity > 98%; IoLiTec Ionic Liquids Technologies GmbH, Heilbronn, Germany) was added separately only to the reference rubber mixture. The same IL was immobilized on the surface of solid supports such as: Ultrasil VN3 silica with specific surface area of 165 m^2^/g, purity > 97% (Evonik Industries, Essen, Germany), silica nanopowder with specific surface area of 560 m^2^/g, particle size of 10–20 nm, purity 99.5% manufactured by Sigma-Aldrich, Schnelldorf, Germany, and carbon black Spheron S0A with specific surface area of 37 m^2^/g produced by Cabot Corporation, Boston, MA, USA. The procedure of DmiBr immobilization on the surface of fillers was described previously [32]. 

The following SILPs were prepared: VN3/IL10, VN3/IL20 (Ultrasil VN3 silica grafted with 10 wt % or 20 wt % of the DmiBr, respectively), nanoSiO_2_/IL10, nanoSiO_2_/IL20 (nanosized silica grafted with 10 wt %, or 20 wt % of the DmiBr, respectively), CB/IL10, and CB/IL20 (carbon black grafted with 10 wt %, or 20 wt % of the DmiBr, respectively). 

### 2.2. Preparation and Characterization of EPDM Compounds

EPDM compounds were prepared in two stages. First, the masterbatch with the composition showed in Table 1 was produced using an internal mixer. This masterbatch contained the fillers (CB and chalk), mineral oil, CaO, and ZnO. The masterbatch was weighed and then cut into several equal parts. Next, the curing system together with pure DmiBr or SILPs was added to each of these pieces using a rolling mill. The general recipes of EPDM composites containing SILPs are listed in Table 2. 

The efficiency of immobilization, understood as the amount of the DmiBr immobilized on the surface, was different for each of the fillers [32]. Therefore, depending on the filler used, different amounts of SILPs were necessary to introduce 3 phr (parts per hundred of rubber) of the DmiBr into the rubber composite (Table 3).

The rheological properties of rubber compounds were examined following the standard PN-ISO 3417:1994. A rotorless curemeter (D-RPA 3000, MonTech, Buchen, Germany) was employed to examine the rheological properties of rubber compounds. The samples were vulcanized at 150 °C using t_95_ and t_80_ (time in which torque increase reaches 95% and 80% of the maximum value, respectively) as vulcanization time. The optimal vulcanization time (t_95_) was determined for the rheometric torque given by Equation (1), where Δ*S* is the torque increase during vulcanization, calculated as the difference between the maximum (*S_max_*) and minimum torque (*S_min_*).

(1) $$S_{95}=0.95\Delta S+S_{min}$$

Using a similar equation, the vulcanization time (t_80_) and the scorch time (t_05_) were determined. Additional rheometric tests were performed at 100 °C to investigate the scorch time (t_05_) in the temperature during rubber compounds processing.

A differential scanning calorimeter DSC1 (Mettler Toledo, Greifensee, Switzerland), previously calibrated with indium and *n*-octane, was used for studying the temperatures and the enthalpy of EPDM curing reactions. During measurements, small pieces of rubber compounds were heated from −100 to 250 °C, with a heating rate of 10 °C/min. Nitrogen (80 mL/min.) was used as the protective gas, whereas liquid nitrogen was applied to cool the sample before the measurement.

A PN-74/C-04236 standard was used to determine the crosslink density of EPDM vulcanizates based on their equilibrium swelling in toluene. The Flory–Rehner equation [33] was applied to calculate the crosslink density with the Huggins parameter of elastomer–solvent (toluene) interaction given by Equation (2) [34], where *V_r_* is the volume fraction of elastomer in swollen gel.

(2) $$\chi =0.425+0.340V_{r}$$

The tensile properties of EPDM vulcanizates were examined following the ISO-37 standard procedures. A Zwick Roell 1435 (Ulm, Germany) universal testing machine was employed to study the tensile strength and elongation at break of dumb-bell specimens with test length of 20 mm and a width of 4 mm.

Disc-shaped samples were used for testing the hardness of EPDM vulcanizates. Measurements were carried out using Shore’s method, following the PN-ISO 868 standard. A Zwick Roell 3105 (Ulm, Germany) hardness tester was used for measurements.

A dynamic mechanical DMA/SDTA861e (Mettler Toledo, Greifensee, Switzerland) analyzer was employed to study the viscoelastic properties of EPDM vulcanizates. Measurements were performed in tension mode using the following conditions: temperature range of −100 to 80 °C (3 °C/min), frequency of 5 Hz, strain amplitude of 10 µm. The glass transition temperature *(T_g_)* was indicated from the peak of the tan δ = f(T) curve, where tan δ is the mechanical loss factor, and T is the measurement temperature.

The thermo-oxidative aging of the vulcanizates was conducted according to the PN-82/c-04216 standard. Vulcanizate plates with a thickness of 2 mm were stored in a drying chamber (BINDER, Tuttlingen, Germany) at 100 °C for 7 days. To estimate the resistance of the vulcanizates to aging, their mechanical properties and crosslink densities were investigated. The aging coefficient (*AF*) was calculated according to Equation (3) [35,36], where TS is the tensile strength of the vulcanizates and EB is the elongation at break:

(3) $$AF=\frac{{\left(EB\times TS\right)}_{after\;aging}}{{\left(EB\times TS\right)}_{before\;aging}}$$

Thermogravimetric analysis (TG) was applied to examine the effect of SILPs on the thermal stability of EPMD vulcanizates. A TGA/DSC1 analyzer (Mettler Toledo, Greifensee, Switzerland), previously calibrated with indium and zinc as standards, was used for measurements. TG analysis was performed using two-step procedure. First, samples were heated in the temperature range of 25–600 °C in an argon atmosphere (flow rate 50 mL/min), with a heating rate of 20 °C/min. Next, the gas was changed into air (flow rate 50 mL/min) and heating was continued up to 900 °C with the same heating rate. 

SEM images of EPDM vulcanizates were taken using an LEO1450 scanning electron microscope (Carl Zeiss AG, Oberkochen, Germany). Prior to the measurement, vulcanizates were frozen in liquid nitrogen and broken down. Next, their fractures were coated with a thin carbon layer and tested.

## 3. Results and Discussion

### 3.1. Cure Characteristics of EPDM Compounds and Crosslink Densities of Vulcanizates

**Figure 1:**
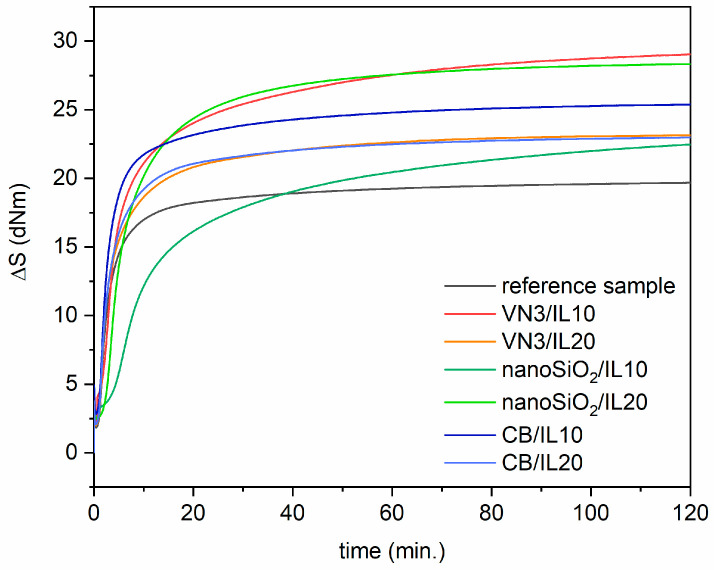
Rheometric curves of EPDM compounds containing SILPs.

Rheometer measurements were gathered to study the influence of SILP materials on the sulfur vulcanization, based on the results of the torque increment during vulcanization, the optimal vulcanization time and scorch time of EPDM compounds. The crosslink densities of EPDM vulcanizates were also determined. From the technological and economic viewpoint, the vulcanization of rubber compounds should quickly occur at the lowest possible temperature. Therefore, the temperature of curing was reduced from the traditionally used 180 °C to 150 °C. To investigate the possibility of reducing the vulcanization time of EPDM compounds at 150 °C, the effect of using t_80_ as the vulcanization time instead of the optimal vulcanization time (t_95_) on the crosslink density of the vulcanizates was studied. No less important was the influence of immobilized DmiBr on the safety of rubber compounds processing, so the scorch time at 100 °C was studied. The rheometric curves of EPDM compounds are presented in Figure 1, whereas the cure characteristics of EPDM compounds and crosslink densities of the vulcanizates are provided in Table 4.

The presented data show that EPDM compounds containing DmiBr immobilized on the surface of fillers exhibited higher minimum torque compared to the EPDM containing pure DMIBr. This parameter corresponds to the viscosity of the uncured rubber compound. As expected, the introduction of an additional portion of fillers in the form of SILPs increases the viscosity of uncured EPDM compounds. Therefore, the values of S_min_ increased with the content of SILPs in the rubber matrix. EPDM compounds containing SILPs showed considerably higher maximum torque, and consequently, higher torque increases during vulcanization compared to the EPDM containing pure DmiBr (Figure 1). The torque increase is an indirect measure of the elastomer crosslinking degree. Higher values of ΔS during vulcanization are correlated with slightly higher crosslink densities of the vulcanizates containing SILP materials. On the other hand, the hydrodynamic effect caused by the introduction of an additional amount of rigid phase in the form of DmiBr-modified fillers into the rubber matrix may have contributed to an increase in the S_max_ and ΔS values. Therefore, despite the highest crosslink density of the vulcanizates crosslinked at t_95_, EPDM-containing DmiBr immobilized on CB exhibited smaller ΔS values compared to rubber compounds with VN3 silica or nanoSiO_2_, which increased the stiffness of the material. 

SILPs prepared using both silicas did not considerably affect the crosslink density of the vulanizates obtained at t_95_ compared to the reference sample containing pure DmiBr. On the other hand, EPDM compounds cured at t_95_, which contain SILPs with CB exhibited considerably higher crosslink density than the reference sample and vulcanizates with both silicas. The lower crosslink density of the vulcanizates containing VN3 or nanoSiO_2_ modified with DmiBr probably resulted from partial adsorption of the curatives, especially the vulcanization accelerator, on the surface of silicas, decreasing the efficiency of crosslinking [18]. The extension of vulcanization time from t_80_ to t_95_ increased the crosslink density of the reference sample and vulcanizates containing DmiBr immobilized on the CB surface. For the EPDM containing DmiBr immobilized on silica, the differences in the crosslink densities of the vulcanizates crosslinked using t_80_ and t_95_ were within the range of measurement error. Considering EPDM crosslinked using t_80_, vulcanizates with SILPs exhibited slighly higher crosslink density than the reference vulcanizate containing pure DmiBr. Analyzing the effect of SILPs on the optimal vulcanization time t_95_, we observed that rubber compounds containing VN3/IL20, nanoSiO_2_/IL20, and DmiBr immobilized on CB exhibited a t_95_ quite similar to the reference sample. EPDM compounds with VN3/IL10 and nanoSiO_2_/IL10 were characterized by a significantly longer t_95_ compared with the reference sample. Most importantly, all rubber compounds containing SILPs showed significantly longer scorch times at 100 °C (26–41 min) or did not cure at this temperature (SILPs containing nanoSiO_2_). Therefore, we confirmed that immobilization of DmiBr on the surface of fillers blocked its activity at the first stage of the vulcanization and enabled the safe processing of rubber compounds at 100 °C. 

Having established the influence of SILPs on the curing characteristics of EPDM compounds as well as the crosslink densities of the vulcanizates, we then employed DSC analysis to study their influences on the temperature and enthalpy of EPDM vulcanization. This method is widely used to study the crosslinking of various polymers [37]. The results for EPDM compounds are presented in Table 5.

Vulcanization of the reference rubber compound with pure DmiBr was a one-step exothermic process occurring in a temperature range of 144–202 °C with an enthalpy of 1.1 J/g, so the amount of heat released was rather low. During this process, sulfur crosslinks (C-S_x_-C) between macromolecules formed, accompanied by cyclic combination of sulfur [38,39]. VN3/IL20, nanoSiO_2_/IL20, and SILPs with CB, significantly decreased the onset temperature and increased the enthalpy of EPDM vulcanization. This effect was the most significant for rubber compounds with VN3/IL20 and nanoSiO_2_/IL20, for which the onset vulcanization temperature was reduced by 64–74 °C and the enthalpy increased by 5–7 J/g. VN3/IL10 and nanoSiO_2_/IL10 did not improve the onset temperature of vulcanization compared to the rubber compound with pure DmiBr. Thus, we concluded that immobilization of 20 wt % DmiBr on the surface of silica is more effective than using 10 wt % of the ionic liquid. During vulcanization, the silanol groups on the silica surface can adsorb accelerators, decreasing the efficiency of vulcanization [18]. Immobilization of DmiBr on the surface of silica blocks the active sites of this filler, consequently decreasing the adsorption of the curing system. The more ionic liquid is immobilized on the surface of silica, the more the active sites on the surface can be blocked. The CB surface has a much lower ability to adsorb the curatives; hence, the amount of DmiBr immobilized on its surface had no significant effects on the temperature and enthalpy of vulcanization. Vulcanization of EPDM compounds containing SILPs with CB occurred in the temperature range of approx. 106–200 °C with enthalpy of approx. 2.0 J/g. DSC analysis of rubber compounds confirmed that SILPs can improve the efficiency of curing process, enabling the temperature reduction of this process, which is important for economic reasons.

### 3.2. Mechanical Properties and Hardness of EPDM Vulcanizates

Crosslink density has a significant influence on the mechanical properties of vulcanizates. From the presented data, SILP materials containing VN3 silica and nanoSiO_2_ slightly affected the amount of crosslinks in the elastomer network compared to the reference vulcanizate with pure DmiBr. On the other hand, considerably higher crosslink densities in comparison with the reference sample were achieved for vulcanizates obtained at t_95_, which contain SILPs with CB. Therefore, their application can influence the tensile properties and hardness of the vulcanizates. Consequently, the effects of SILPs on the mechanical properties and hardness of EPDM vulcanizates were examined. Studies were performed for the EPDM vulcanizates obtained at t_80_ and t_95_. The results are presented in Table 6.

The data compiled in Table 6 show that, regardless of whether t_80_ or t_95_ were used as vulcanization times, EPDM vulcanizates with SILP materials exhibited approx. 0.5–1.9 MPa higher SE_100_ modulus and about 1–4 ShA higher hardness compared with the reference vulcanizate containing pure DmiBr. This resulted mainly from the addition of 17–35 phr of active fillers, such as silica and CB in the form of SILPs. The hardness of EPDM containing SILPs was in the range of 67–73 ShA, so this material is still a medium hardness rubber.

Addition of SILPs affected the tensile strength and elongation at break of the vulcanizates. Reference vulcanizate obtained at t_80_ exhibited a tensile strength of 10.7 MPa and elongation at break of 437%. Vulcanizates prepared at t_80_ containing DmiBr-modified fillers demonstrated slightly higher tensile strength (by approx. 1 MPa) and lower elongation at break, in the range of 289%–374%. As mentioned above, the crosslink densities of the vulcanizates with SILPs and pure DmiBr were comparable, so the effect of SILPs on the tensile strength and elongation at break of the vulcanizates can result from the addition of active fillers, which increase the stiffness and reduce the elasticity of the vulcanizates. Notably, that lower elongation at break exhibited vulcanizates with a higher amount of SILPs, thus containing 10 wt % of immobilized DmiBr. Regarding the tensile strength and elongation at break of the EPDM composites cured at t_95_, similar effect of SILPs was observed. DmiBr-modified fillers increased the tensile strength by approx. 1 MPa and reduced the elongation at break from 361% for the reference sample to 232%–325% for SILP-containing vulcanizates. Prolonging the vulcanization time from t_80_ to t_95_ increased the SE_100_ modulus and hardness of the vulcanizates, and significantly reduced their elongation at break due to higher crosslink density of the vulcanizates. A less pronounced effect of the vulcanization time was observed for the tensile strength.

### 3.3. Dynamic Mechanical Properties of EPDM Vulcanizates

**Figure 2:**
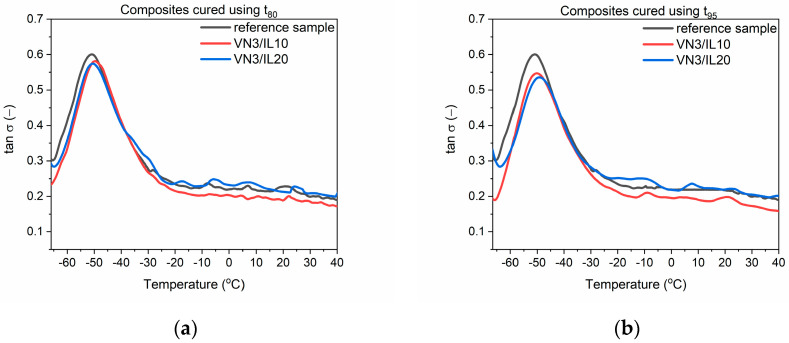
Mechanical loss factor (tan δ) as a function of temperature for EPDM vulcanizates with SILPs containing VN3: (**a**) vulcanizates obtained at t_80_, (**b**) vulcanizates obtained at t_95_.

**Figure 3:**
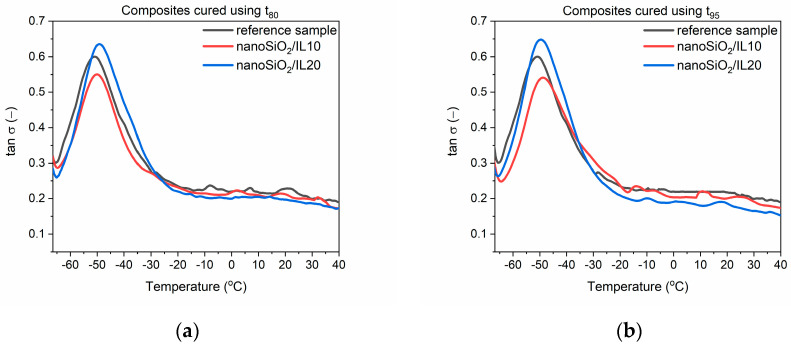
Mechanical loss factor (tan δ) as a function of temperature for EPDM vulcanizates with SILPs containing nanoSiO_2_: (**a**) vulcanizates obtained at t_80_, (**b**) vulcanizates obtained at t_95_.

**Figure 4:**
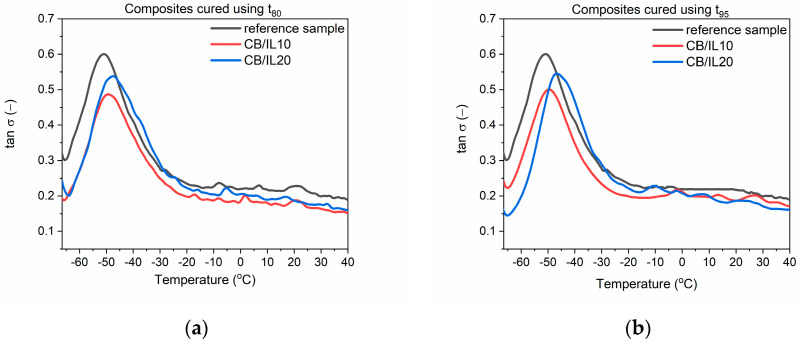
Mechanical loss factor (tan δ) as a function of temperature for EPDM vulcanizates with SILPs containing CB: (**a**) vulcanizates obtained at t_80_, (**b**) vulcanizates obtained at t_95_.

Dynamic mechanical properties are very important for technological applications of EPDM rubber products, which often are subjected to variable stresses or deformations during their operation (window and door seals, O-rings, gaskets, or car bumpers). The largest market for EPDMs is the automotive industry, so the ability of elastomer products to suppress vibrations is crucial considering their application as shock absorbers or elements of vibration dampeners. DMA analysis was applied to examine the influence of SILP materials on the mechanical loss factor (tan δ), which corresponds to the ability of the material to suppress vibration. Tan δ is determined as the ratio of the material loss modulus (E”) to its storage modulus (E’) [40]. The mechanical loss factor was measured as a function of temperature to study the influence of SILPs on the glass transition (T_g_) of EPDM and its dynamic mechanical properties in the glassy state and in the rubbery elastic region. The results for EPDM vulcanizates cured at t_80_ and t_95_ are presented in Table 7 and Figure 2, Figure 3 and Figure 4.

First, the influence of SILPs on the glass transition temperature (T_g_) of EPDM was investigated. T_g_ is a temperature below which the segmental and chain mobility in the elastomer disappears; the material is brittle and unable to transfer stress. This temperature can be determined as a temperature of the peak tan δ in the DMA curve [41].

The T_g_ of EPDM was approximately −50.9 °C and −48.8 °C for the reference vulcanizates, with pure DmiBr obtained at t_80_ and t_95_, respectively. In the case of vulcanizates prepared using t_95_, addition of SILP materials did not significantly influence the temperature at which the elastomer undergoes transition from a hard, glassy state to a rubbery state. Regarding vulcanizates obtained at t_80_, SILPs containing both silicas did not significantly affect the T_g_, whereas vulcanizates with DmiBr-modified CB exhibited approx. 3 °C higher T_g_ compared to the reference sample with pure DmiBr. Prolonging the vulcanization time from t_80_ to t_95_ increased the T_g_ by 2 °C only for the reference vulcanizate. The T_g_ values of SILP-containing EPDM were comparable considering the experimental error. Hence, from a technological point of view, SILPs or vulcanization at t_80_ should not affect the operating temperature range of EPDM products.

EPDM composites cured at t_80_ and t_95_ exhibited similar values of the mechanical loss factor both at T_g_ and in the rubbery elastic region. Regardless of the vulcanization time used, SILPs containing VN3 or nanosized silica did not significantly affect the tan δ values at T_g_ or in the rubbery elastic state compared to the reference sample containing pure DmiBr. EPDM vulcanizates with DmiBr-modified CB exhibited slightly lower values of tan δ than other vulcanizates. However, considering the magnitude of the tan δ changes, SILPs should not have a significant impact on the material’s ability to dampen vibrations. Vulcanizates with SILPs exhibited stable dynamic mechanical properties in the rubbery elastic region since the values of tan δ did not change considerably with increasing temperature. This is essential for potential applications of EPDM composites.

### 3.4. Thermo-Oxidative Aging of EPDM Vulcanizates

**Figure 5:**
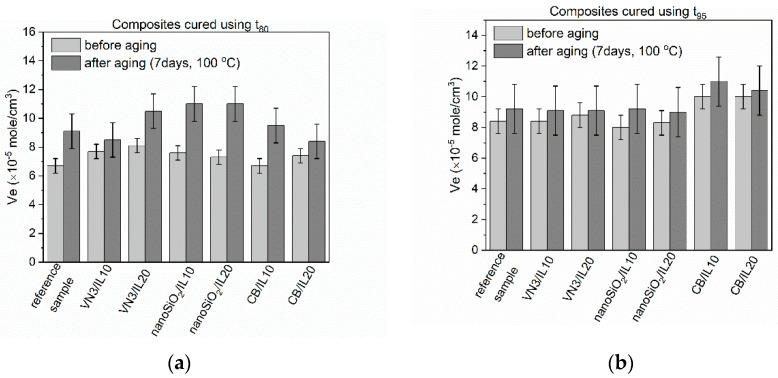
Effect of thermo-oxidative aging on the crosslink density of EPDM vulcanizates containing SILPs: (**a**) vulcanizates cured using t_80_; (**b**) vulcanizates cured using t_95_.

**Figure 6:**
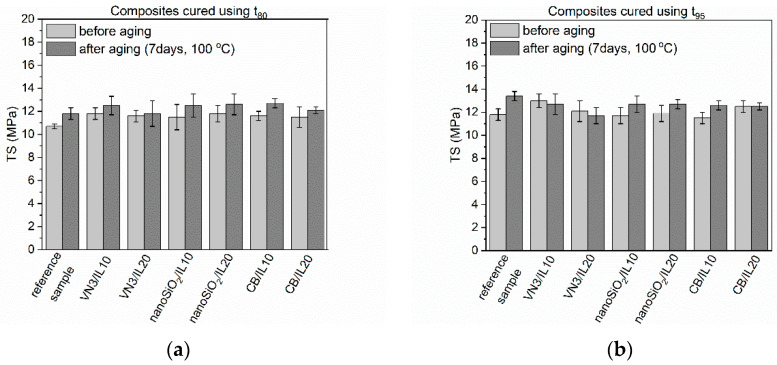
Effect of thermo-oxidative aging on the tensile strength of EPDM vulcanizates containing SILPs: (**a**) vulcanizates cured using t_80_; (**b**) vulcanizates cured using t_95_.

**Figure 7:**
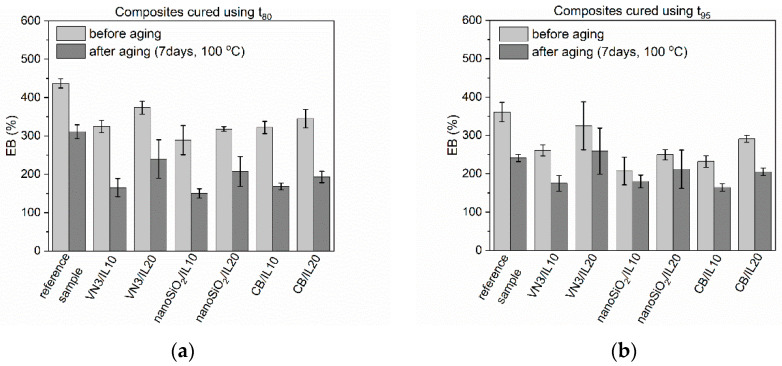
Effect of thermo-oxidative aging on elongation at break of EPDM vulcanizates containing SILPs: (**a**) vulcanizates cured using t_80_; (**b**) vulcanizates cured using t_95_.

**Figure 8:**
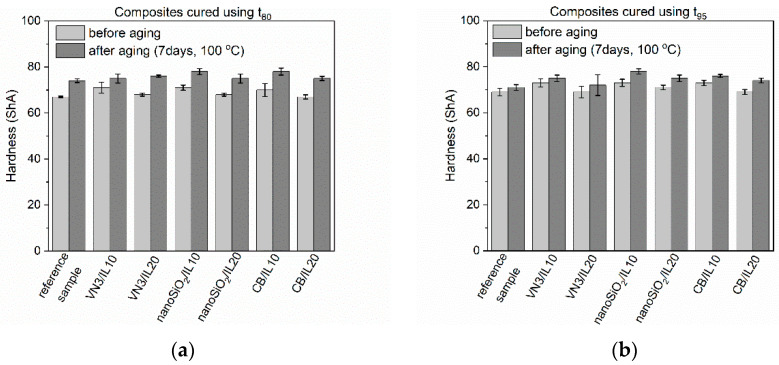
Effect of thermo-oxidative aging on the hardness of EPDM vulcanizates containing SILPs: (**a**) vulcanizates obtained at t_80_, (**b**) vulcanizates obtained at t_95_.

Rubber products are often exposed to prolonged exposure to elevated temperatures. Generally, EPDM elastomers have high heat and aging resistance. For application reasons, these properties of SILPs should not deteriorate. Thermo-oxidative aging of the vulcanizates was performed at 100 °C for seven days. Next, their crosslink densities as well as mechanical properties were examined and compared with the values obtained for non-aged vulcanizates. Studies were performed for vulcanizates obtained using both t_80_ and t_95_ as the vulcanization times. The results are presented in Figure 5, Figure 6, Figure 7 and Figure 8 and in Table 8.

EPDM vulcanizates, especially those cured at t_80_, exhibited higher crosslink densities after prolonged exposure to 100 °C. Thus, thermo-oxidative aging initiated further curing of the elastomer (Figure 5). However, this effect was much less pronounced for the vulcanizates obtained at t_95_, due to their higher crosslink density before the aging process. A similar influence of thermo-oxidative aging on the crosslink density of the vulcanizates has been reported for other elastomers, such as SBR, natural rubber, and acrylonitrile butadiene elastomer [16,42,43,44]. Regarding the vulcanizates cured at t_80_, a slightly higher increase in crosslink density demonstrated vulcanizates containing SILPs, especially based on VN3 and nanosized silica. In the case of t_80_ crosslinked EPDM, the largest increase in crosslink density during thermo-oxidative aging was observed for vulcanizates with SILPs containing nanoSiO_2_.

Thermo-oxidative aging increased by 1–2 MPa the tensile strength of SILPs containing vulcanizates obtained at t_80_ compared to vulcanizates before aging. A slight improvement in TS was also achieved (by approx. 1 MPa) for vulcanizates cured at t_95_. Similar changes in tensile strength were observed for the reference vulcanizates regardless of their vulcanization time. The elongation at break of the vulcanizates obtained at t_80_ was reduced by about 110%–160% due to the increase in their crosslink density (Figure 7). A smaller reduction in elongation at break showed vulcanizates with SILPs, especially nanoSiO_2_, cured at t_95_. This resulted from a smaller increase in the crosslink density of these vulcanizates compared to EPDM cured at t_80_.

The hardness of EPDM vulcanizates cured at t_80_ (Figure 8), which were subjected to thermo-oxidative aging, increased by approx. 6–10 ShA due to the increase in their crosslink density. This effect was less pronounced for SILP-containing vulcanizates obtained using t_95_.

Based on the changes in the mechanical properties of vulcanizates due to the aging process, the AF coefficient was calculated (Table 8). The higher AF corresponds to the smaller changes in the TS and EB values of the vulcanizates resulting from the aging process, and consequently, the better aging resistance of the material. EPDM vulcanizates are quite resistant to thermo-oxidative aging at 100 °C. Regardless of the vulcanization time, the AF for the reference vulcanizate with pure DmiBr was 0.8. SILPs containing vulcanizates cured at t_80_ demonstrated lower AF (0.5–0.7) compared to the reference samples. Thus, the use of an anti-aging agent should be considered. On the other hand, SILPs did not significantly affect the resistance to thermo-oxidative aging of the vulcanizates obtained at t_95_ compared to the reference vulcanizate with pure DmiBr. The AF values for these vulcanizates were in the range of 0.7–0.9.

### 3.5. Dispersion of Curatives, Filler, and SILPs in EPDM Matrix

**Figure 9:**
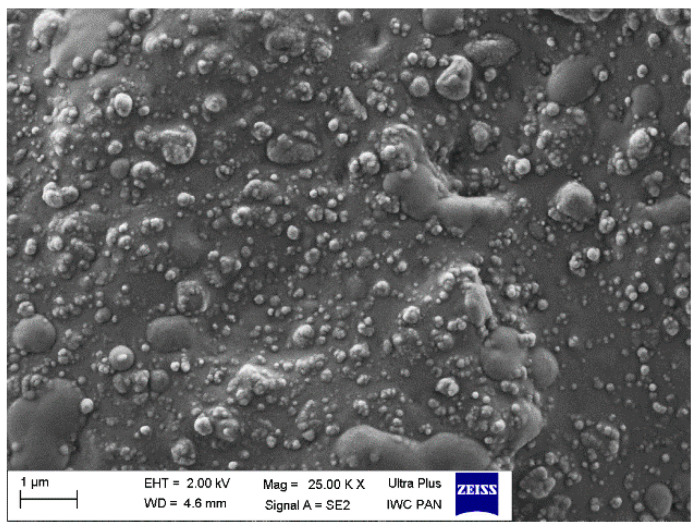
Scanning electron microscopy (SEM) image of the reference vulcanizate containing pure DmiBr.

**Figure 10:**
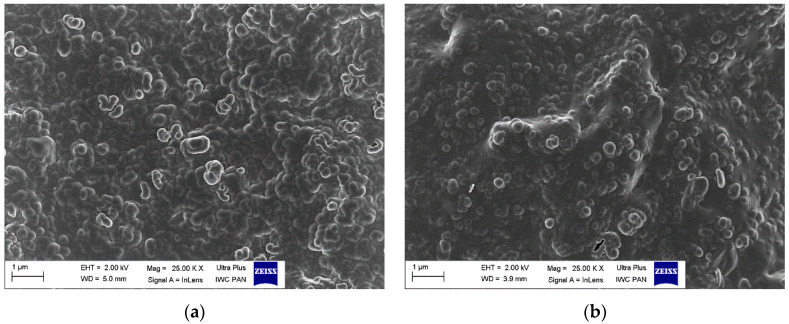
SEM images of EPDM vulcanizates containing: (**a**) VN3/IL10; (**b**) VN3/IL20.

**Figure 11:**
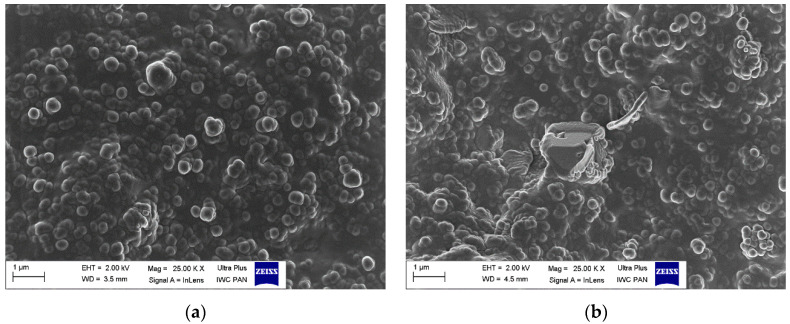
SEM images of EPDM vulcanizates containing: (**a**) nanoSiO_2_/IL10; (**b**) nanoSiO_2_/IL20.

**Figure 12:**
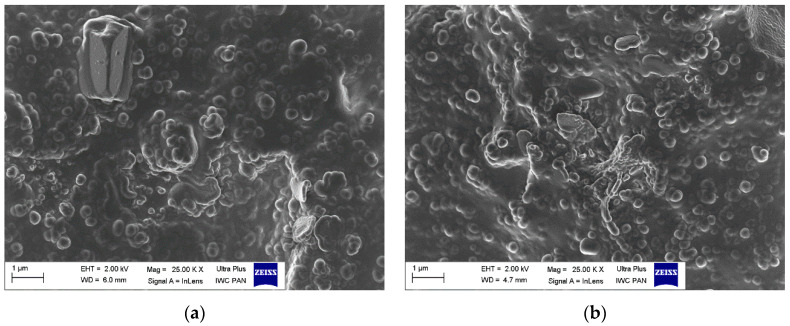
SEM images of EPDM vulcanizates containing: (**a**) CB/IL10; (**b**) CB/IL20.

The main goal of elastomer technology is to produce a rubber product with the appropriate mechanical performance and hardness; therefore, reinforcing additives are applied [42]. A significant aspect of the reinforcement effect is producing a uniform dispersion of the filler particles in the elastomeric matrix, which results in a good interphase adhesion. On the other hand, the contact between components of the curing system in the elastomer matrix should be maximized to improve the degree of crosslinking. Most solid additives exhibit a high agglomeration ability in the elastomer matrix; therefore, achieving homogenous distribution of their particles in the elastomer is technologically difficult. Introducing an additional amount of fillers in the form of SILPs may hinder the production of vulcanizates characterized by homogeneous dispersion of individual ingredients. The agglomerates may concentrate stresses when vulcanizates undergo external deformations resulting in deterioration of mechanical properties. Therefore, SEM images were captured to study the dispersion degree of solid ingredients in the EPDM matrix containing SILPs. The results are shown in Figure 9, Figure 10, Figure 11 and Figure 12.

In our previous work, we proved that the particles of VN3/IL20 and CB/IL20 were quite uniformly distributed in the EPDM matrix and demonstrated good adhesion to the elastomer [32]. In this work, SEM images were captured for vulcanizate with pure DmiBr as well as for EPDM composites containing other SILPs. In the case of a reference vulcanizate with pure DmiBr, the particles of fillers (CB and chalk) as well as other solid additives (CaO, ZnO, curatives) were uniformly distributed and quite well wetted by the elastomer matrix (Figure 9). Regarding the vulcanizates with DmiBr-modified VN3 silica, the dispersion of particles in the EPDM matrix was even better than for the reference vulcanizate and no agglomeration was observed (Figure 10). The homogeneously dispersed particles exhibited very good adhesion to the elastomer, being surrounded by an elastomeric film that penetrated between them. Particles of SILPs containing nanoSiO_2_ or CB were also homogeneously dispersed in the EPDM and exhibited good wettability by elastomer matrix (Figure 11 and Figure 12). Only individual agglomerates of approx. 1 µm embedded in the elastomer matrix were seen for nanoSiO_2_/IL20 and CB/IL10. The amount of SILPs in the rubber compounds did not affect their dispersion in the elastomer. The most important is that incorporation of an additional amount of fillers in the form of SILPs did not deteriorate the dispersion of solid additives in the EPDM matrix. Moreover, the phase adhesion of the solid particles to the elastomer matrix seems to be much better in the case of SILP-containing composites. The particles appear to be deeply embedded within the elastomer matrix and completely covered with a rubber film. The fracture of vulcanizates containing SILPs appear to be smoother than the samples with a pure DmiBr. Therefore, it was concluded that immobilization of DmiBr on the surface of fillers improved their wettability, and consequently phase adhesion to the elastomer matrix.

### 3.6. Thermal Stability of EPDM Vulcanizates

**Figure 13:**
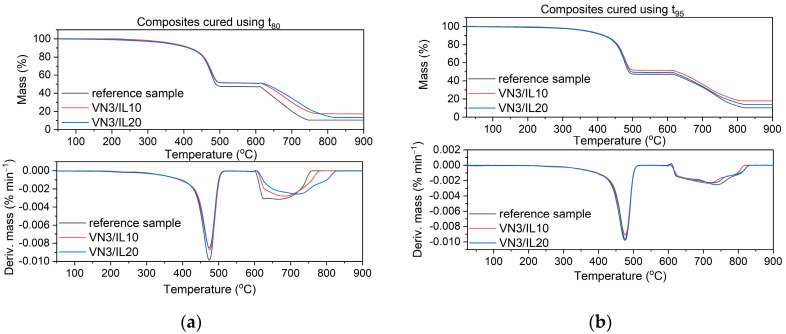
Thermogravimetric (TG) and differential thermogravimetric (DTG) curves of EPDM vulcanizates with SILPs containing VN3: (**a**) vulcanizates obtained at t_80_, (**b**) vulcanizates obtained at t_95_.

**Figure 14:**
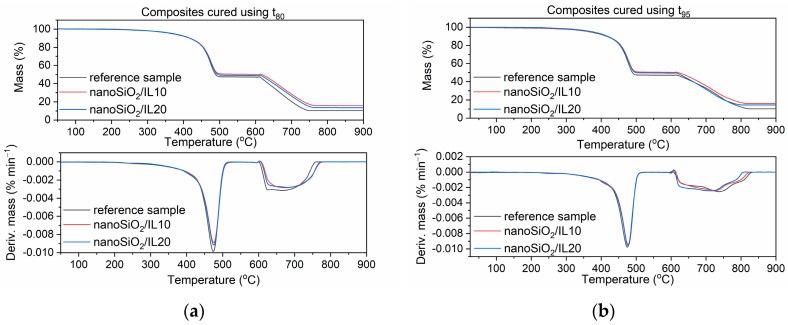
TG and DTG curves of EPDM vulcanizates with SILPs containing nanoSiO_2_: (**a**) vulcanizates obtained at t_80_, (**b**) vulcanizates obtained at t_95_.

**Figure 15:**
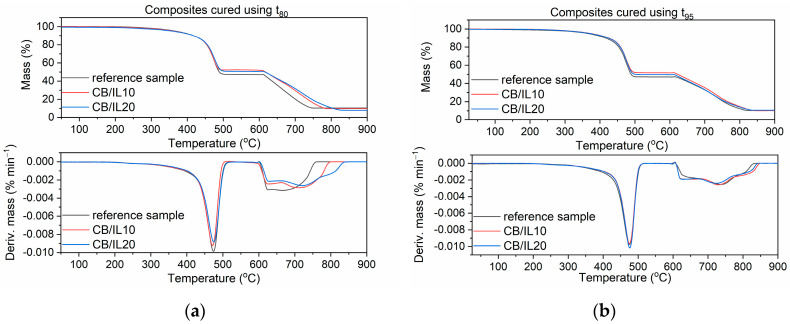
TG and DTG curves of EPDM vulcanizates with SILPs containing CB: (**a**) vulcanizates obtained at t_80_, (**b**) vulcanizates obtained at t_95_.

Thermal stability is crucial for rubber products. Therefore, TG analysis was conducted to investigate the effect of SILPs on the thermal stability of EPDM composites. The temperature at 5% mass change of sample (T_5%_) was taken as the onset temperature of thermal degradation. The temperature of the peak of the differential thermogravimetric (DTG) curves (T_DTG_) was determined as the temperature of elastomer decomposition. The results are provided in Table 9 and Figure 13, Figure 14 and Figure 15.

During the first step of TG measurement, samples were heated in the temperature range of 25–600 °C in an argon atmosphere. Mass losses at this range of temperature correspond to pyrolysis of the elastomer and thermal degradation of organic ingredients, such as vulcanization accelerators, plasticizer, and DmiBr. Since the contents of elastomer and organic additives were the same for each of the samples, the mass loss in this temperature range was similar for all vulcanizates; the differences were within the range of experimental error. At temperatures above 600 °C, samples were heated in air, so the mass losses resulted from burning of the carbon black and the thermal decomposition of the chalk. These mass losses were the highest for the vulcanizates with CB/IL10 and CB/IL20, which both resulted from the combustion of carbon black, which was used as a filler in EPDM compounds and the additional amount that was introduced in the form of SILPs. Regarding the reference vulcanizate, the mineral residue at 900 °C consisted of ash, ZnO, and CaO used as desiccant and formed during thermal decomposition of the chalk. Vulcanizates with SILPs containing VN3 silica or nanoSiO_2_ exhibited higher residues at 900 °C compared with the reference vulcanizate and samples containing DmiBr-modified CB. This resulted from the presence of silica, which did not decompose (apart from losing some water adsorbed as a moisture) and remained as a residue after the thermal decomposition of the vulcanizate. 

The onset decomposition temperature, T_5%_, determined for vulcanizate containing pure DmiBr was 369 °C, regardless of the vulcanization time used. EPDM compounds containing SILPs with nanoSiO_2_ showed an approx. 4 °C higher T_5%_ temperature. SILPs containing VN3 silica did not affect the thermal stability of the vulcanizates, whereas a slight deterioration of the T_5%_ temperature was observed for the vulcanizates containing DmiBr-modified CB, which were obtained at t_80_. SILPs did not significantly influence the T_DTG_ values, nor the temperature of elastomer pyrolysis, which led to major decomposition of the vulcanizates. Therefore, we concluded that SILPs did not reduce the thermal stability of EPDM composites, which are thermally stable up to a temperature of 360 °C. Prolonging the vulcanization time from t_80_ to t_95_ did not affect the T_5%_ temperature of the reference vulcanizate and vulcanizates with SILPs containing both silicas. In the case of SILPs with CB, vulcanizates obtained at t_95_ exhibited an approx. 9–12 °C higher T_5%_ temperature due to a significantly higher crosslink density compared to the vulcanizates cured at t_80_. No considerable influence of the vulcanization time of EPDM compounds on the T_DTG_ values was observed.

## 4. Conclusions

The influence of DmiBr ionic liquid immobilized on the surface of fillers, such as silica and carbon black, on the vulcanization process, mechanical performance, and thermal behavior of EPDM elastomer was investigated.

SILPs consisted of DmiBr immobilized on the surface of VN3 and nanoSiO_2_ silica as well as on carbon black, enabling the control the sulfur vulcanization of EPDM compounds and resulting in the reduction of the optimal vulcanization time at a reduced temperature of this process as well as a suitably long scorch time, which is important for the safe processing of rubber compounds.

Immobilization of DmiBr on the surface of both silicas and carbon black blocks its activity in crosslinking at 100 °C, which enables safe processing of EPDM compounds at this temperature. Rubber compounds containing SILPs with nanoSiO_2_ did not crosslink at 100 °C, whereas EPDM containing IL immobilized onto VN3 silica exhibited a scorch time at 100 °C of approx. 27 min. (VN3/IL10) and 40 min. (VN3/IL20), respectively. The optimal time of vulcanization at 150 °C for rubber compounds containing SILPs with 20 wt % immobilized DmiBr was comparable to the reference composite with pure ionic liquid. SILPs based on carbon black exhibited a scorch time at 100 °C of about 26 min and a t_95_ at 150 °C comparable to the reference rubber compound.

Regarding vulcanizates obtained at t_95_, SILPs containing both silicas do not considerably affect the crosslink density compared with the reference vulcanizate containing pure DmiBr. Vulcanizates containing SILPs with CB exhibit higher crosslink density than the reference sample. Most SILPs, especially that containing 20 wt % DmiBr, significantly decreased the onset vulcanization temperature and intensified this process. This confirms the catalytic activity of SILPs in the interfacial crosslinking reactions.

Vulcanizates containing DmiBr immobilized on the surface of fillers demonstrated slightly higher tensile strength and lower elongation at break, as well as slightly higher hardness in comparison with the reference vulcanizate. These resulted mainly from the additional amount of active fillers introduced into the elastomer matrix in the form of SILPs. Considering the magnitude of the loss factor changes, we concluded that SILPs do not significantly impact the ability of EPDM vulcanizates to dampen vibrations. Vulcanizates with SILPs demonstrate stable dynamic properties in the rubbery elastic region.

Thermo-oxidative aging causes further crosslinking of EPDM elastomer and consequently an increase in tensile strength accompanied by a considerable reduction in the elongation at break of the vulcanizates. SILPs reduce the resistance to thermo-oxidative aging of EPDM vulcanizates cured at t_80_. The use of an anti-aging agent should be considered. On the other hand, SILPs do not affect the aging resistance of the vulcanizates obtained using t_95_ as the vulcanization time.

Regardless of the vulcanization time used, DmiBr immobilized on the surface of silica or CB does not affect the thermal stability of EPDM composites, which are thermally stable up to a temperature of 360 °C.

## Figures and Tables

**Table 1:** General formula of the ethylene–propylene–diene rubber (EPDM) masterbatch, parts per hundred of rubber (phr).

Ingredient	Masterbatch
EPDM	100.0
Carbon black	125.0
Mineral oil	65.0
Chalk	40.0
CaO	7.5
ZnO	7.5
Stearic acid	1.5

**Table 2:** General formula of EPDM compounds containing supported ionic liquid phases (SILPs), phr; MBTS, benzothiazole disulfide; ZDBC, zinc bis(dibutyl dithiocarbamate); DPG, 1,3-diphenylguanidine; DmiBr, 1-decyl-3-methylimidazolium bromide.

Ingredient	Reference Sample	EPDM Compounds
Masterbatch	346.5 ^a^	346.5
Sulfur	1.5	1.5
MBTS	1.0	1.0
ZDBC	2.5	2.5
DPG	0.3	0.6
Vulkalent	0.5	0.5
DmiBr	3.0	-
SILPs	-	^b^

^a^ 346.5 phr of masterbatch contains 100 phr of EPDM rubber, ^b^ the type and content of SILPs is given in Table 3.

**Table 3:** Type and amount of SILPs in EPDM compounds.

Vulcanizate	SILP Material	Amount of SILP in EPDM Compounds (phr)
EPDM 1	VN3/IL10	35.1
EPDM 2	VN3/IL20	17.4
EPDM 3	nanoSiO_2_/IL10	29.2
EPDM 4	nanoSiO_2_/IL20	19.2
EPDM 5	CB/IL10	34.0
EPDM 6	CB/IL20	18.7

**Table 4:** Cure characteristics of EPDM compounds at 150 °C and crosslink densities of vulcanizates (ΔS, torque increase; t_05_, scorch time; t_80_, t_95_, vulcanization times; ν_e_, crosslink density. Standard deviations: S_min_ ± 0.4 dNm, S_max_ ± 3.5 dNm, ΔS ± 2.9 dNm, t_05_ ± 2.4 min, t_80_ ± 1.7 min., t_95_ ± 1.6 min, ν_e_ ± 0.9 × 10^−5^ mole/cm^3^).

EPDM Compounds	S_min_ (dNm)	S_max_ (dNm)	ΔS (dNm)	t_05_ at 100 °C (min)	t_80_ (min)	t_95_ (min)	ν_e_ (× 10^−5^ mole/cm^3^)	
Reference sample	1.8	19.7	17.9	9	9	35	6.7 *	8.4 **
VN3/IL10	2.5	29.5	27.0	41	20	65	7.7	8.4
VN3/IL20	1.9	23.1	21.2	27	11	41	8.1	8.8
nanoSiO_2_/IL10	2.8	22.5	19.7	no curing	35	84	7.6	8.0
nanoSiO_2_/IL20	2.3	28.4	26.1	no curing	15	39	7.3	8.3
CB/IL10	2.8	25.4	22.6	26	8	39	6.9	10.0
CB/IL20	2.1	23.0	20.9	27	9	37	7.4	10.0

Note: * crosslink density of the vulcanizate obtained using t_80_, ** crosslink density of the vulcanizate obtained using t_95._

**Table 5:** Temperature and enthalpy of EPDM vulcanization determined by differential scanning calorimetry (DSC) (standard deviations: temperature ± 13.0 °C; enthalpy of vulcanization (ΔH) ± 2.6 J/g).

EPDM Compounds	Temperature of Vulcanization (°C)	ΔH (J/g)
Reference sample	144–202	1.1
VN3/IL10	155–244	2.2
VN3/IL20	80–219	6.1
nanoSiO_2_/IL10	142–223	4.1
nanoSiO_2_/IL20	70–179	7.9
CB/IL10	110–201	1.4
CB/IL20	106–203	2.5

**Table 6:** Mechanical properties and hardness of EPDM vulcanizates (SE_100_, modulus at a relative elongation of 100%; TS, tensile strength; EB, elongation at break; H, hardness. Standard deviations: SE_100_ ± 0.7 MPa, TS ± 0.4 MPa, EB ± 47%, H ± 4 ShA).

EPDM Vulcanizate	SE_100_ (MPa)		TS (MPa)		EB (%)		H (ShA)	
	t_80_	t_95_	t_80_	t_95_	t_80_	t_95_	t_80_	t_95_
Reference sample	3.1	4.0	10.7	11.8	437	361	67	69
VN3/IL10	4.8	5.9	11.8	13.0	325	261	71	73
VN3/IL20	3.6	4.2	11.6	12.1	374	325	68	69
nanoSiO_2_/IL10	4.8	5.8	11.5	11.7	289	207	71	73
nanoSiO_2_/IL20	4.4	5.5	11.8	11.9	318	250	68	71
CB/IL10	5.0	5.9	11.6	11.5	322	232	70	73
CB/IL20	4.0	4.9	11.5	12.5	345	291	67	69

**Table 7:** Glass transition temperature (T_g_) and mechanical loss factor (tan δ) of EPDM vulcanizates (standard deviations: T_g_ ± 1.1 °C, tan δ ± 0.2).

EPDM Vulcanizate	T_g_ (°C)		tan δ at T_g_ (-)		tan δ at 25 °C (-)		tan δ at 40 °C (-)	
	t_80_	t_95_	t_80_	t_95_	t_80_	t_95_	t_80_	t_95_
Reference sample	−50.9	−48.8	0.60	0.62	0.21	0.17	0.21	0.16
VN3/IL10	−49.6	−48.9	0.58	0.55	0.19	0.18	0.18	0.17
VN3/IL20	−50.5	−49.6	0.58	0.56	0.21	0.21	0.20	0.19
nanoSiO_2_/IL10	−50.0	−48.9	0.56	0.55	0.20	0.20	0.18	0.18
nanoSiO_2_/IL20	−49.5	−48.4	0.64	0.63	0.19	0.18	0.18	0.16
CB/IL10	−48.5	−47.6	0.49	0.51	0.18	0.19	0.15	0.16
CB/IL20	−47.4	−47.1	0.54	0.56	0.18	0.19	0.16	0.16

**Table 8:** Aging coefficient (AF) of EPDM vulcanizates (SD ± 0.1).

EPDM Compounds	AF (-)	
	t_80_	t_95_
Reference vulcanizates	0.8	0.8
VN3/IL10	0.5	0.7
VN3/IL20	0.7	0.8
nanoSiO_2_/IL10	0.6	0.9
nanoSiO_2_/IL20	0.7	0.9
CB/IL10	0.6	0.8
CB/IL20	0.6	0.8

**Table 9:** Onset temperature of thermal degradation (T_5%_), differential thermogravimetric (DTG) peak temperature (T_DTG_), and total mass loss (Δm) during thermal degradation of EPDM composites (standard deviations: T_5%_ ± 4.1 °C; T_DTG_ ± 8.0 °C; Δm ± 3.4).

EPDM Vulcanizates	T_5%_ (°C)		T_DTG_ (°C)		Δm (25–600 °C) (%)		Δm (600–900 °C) (%)		Residue at 900 °C (%)	
	t_80_	t_95_	t_80_	t_95_	t_80_	t_95_	t_80_	t_95_	t_80_	t_95_
Reference sample	369	369	477	473	51.2	51.8	36.8	36.9	12.0	11.6
VN3/IL10	369	372	476	478	49.5	48.4	35.5	33.5	15.0	18.1
VN3/IL20	369	371	474	477	49.1	50.9	37.7	35.2	13.2	13.9
nanoSiO_2_/IL10	373	372	475	478	50.5	49.5	34.2	34.2	15.3	16.3
nanoSiO_2_/IL20	372	374	475	475	51.2	50.5	35.1	34.9	13.7	14.6
CB/IL10	365	374	472	477	48.9	48.2	42.1	41.5	9.0	10.3
CB/IL20	360	372	474	476	49.0	50.1	42.5	40.0	8.5	9.9
